# Emergence of *IntI1* associated *bla*_VIM-2_ gene cassette-mediated carbapenem resistance in opportunistic pathogen *Pseudomonas stutzeri*

**DOI:** 10.1038/emi.2017.12

**Published:** 2017-05-10

**Authors:** Sabrin Bashar, Santonu Kumar Sanyal, Munawar Sultana, M Anwar Hossain

**Affiliations:** 1 Department of Microbiology, University of Dhaka, Dhaka 1000, Bangladesh

**Dear Editor,**

Carbapenems are considered as the last resort of antibiotic for the treatment of infection but many Gram-negative organisms have developed resistance to this antibiotic through loss or alteration of outer membrane porin protein OprD, over-expression of efflux pump, hyperproduction of an AmpC-type-β-lactamase and/or carbapenemase.^1^ Carbapenemases are β-lactamases with catalytic efficiencies for carbapenem hydrolysis, including enzymes from Ambler’s classes A (extended spectrum β-lactamase), B (metallo-β-lacatamase, MBL) and D (Oxacillinases, OXA). The serine carbapenemases are derivatives of class A (e.g., IMI, KPC, GES) and class D enzymes (e.g., OXA-23, OXA-40, OXA-48) that hydrolyze carbapenems poorly but able to confer resistance. MBLs (e.g., IMP, VIM, GIM, SPM-l) can hydrolyze all β-lactams, including carbapenems (with the exception of aztreonam).^1, 2^ Dutch imipenemase (DIM-1), a novel subclass 1 MBL can significantly hydrolyze broad-spectrum cephalosporins and carbapenems.^3^ IMP and Verona integron-encoded metallo-β-lactamase (VIM) derivatives are widespread MBLs^4^ and can be harbored within gene cassettes embedded into class 1 integron structure^3^ as we know integrons can act as expression vector for the genes captured in the cassette.^5^

*Pseudomonas stutzeri* is considered as environmental pseudomonad species and rarely cause nosocomial infection.^6^ IMP and VIM derivatives have been identified in *P. stutzeri*,^7^ but their integration in integron gene cassettee has not been reported. DIM-1 embedded in a class 1 integron located on a 70 kb plasmid has been reported originally in *P. stutzeri*,^3^ however, subsequently identified in other species and locations.^8^ In this study, we report the presence of VIM-2 MBL along with retrieval of associated gene cassette in clinical *P. stutzeri* isolate. A report is presented here concerning association of multidrug-resistant (MDR) clinical *P. stutzeri* 40D2 (initially named as 40/D/Mac2) isolated from the pus sample of a diabetic patient with a deep tissue foot infection (cellulitis) in a tertiary care hospital in Dhaka, Bangladesh.^9^ Initially, 20 isolates were recovered among which three carbapenem resistant *Pseudomonas* isolates (40/D/Mac2, 40/D/Swab1, 40/D/CIP+Cefo1) were retrieved belonging to a single genotype, detected through amplified ribosomal DNA restriction analysis. Isolate 40D2 was selected for detailed investigation.

Isolate 40D2 was fast growing, colony appeared as pale, irregular shaped, wrinkled, gummy and flat in Nutrient Agar medium (Oxoid, UK) and microscopically Gram-negative and rod shaped bacterium. The 16S rRNA gene amplification, sequencing (GenBank accession number KT716345) and phylogenetic analysis revealed that the isolate was identified as *P. stutzeri* (accession number KP202687.1) with 100% sequence identity.

The antibiotic susceptibility was assessed by standard disc diffusion and microdilution methods,^10^ which demonstrated that the isolated *P. stutzeri* 40D2 was MDR, being resistant to imipenem (MIC 256 mg/L), meropenem, doripenem, nitrofurantoin, ampicillin, oxacillin, gentamicin, trimethoprim, ciprofloxacin, levofloxacin, nalidixic acid, cefalexin, cefuroxime, cefotaxime, cefepime and surprisingly aztreonam. The isolate showed reduced susceptibility to azithromycin and chloramphenicol and was susceptible to amikacin, polymyxin B and colistin. Plasmid isolation was done by both Alkaline Lysis method ^11^ and Minipreps plasmid DNA Purification kit (Wizard Plus SV, Promega, Madison, WI, USA). Both methods were able to retrieve plasmids of control strain *Escherichia coli* V517 (plasmid size 2.2–55.5 kb); however no plasmid of these size range was retrieved from *P. stutzeri* 40D2 under our experimental condition. Carbapenemase production was confirmed by Blue-Carba Test,^12^ and MBL production was determined by combined disc test method.^13^ The occurrence of MBLs (*bla*_VIM-2-like_*, bla*_NDM-1-like_, *bla*_IMP-3-like_, *bla*_IMP-4 like_) was analyzed by PCR (Supplementary Table S1) revealing the presence of *bla*_VIM-2_ gene and the amplicon sequencing was carried out in order to determine 100% nucleotide sequence identity to that of *Pseudomanas aeruginosa* strain 7052 integron (GenBank accession number AY943084).

Integron class 1 associated gene cassettes were amplified using a total of 10 sets of primers (reference and designed primers, Supplementary Table S1). The amplicons were sequenced and the overlapping sequences were assembled to 4409 nucleotides (Figure 1A, GenBank accession number KT716347). After sequencing and homology analysis of the nucleotide sequences (http://www.ncbi.nlm.nih.gov), the location of *bla*_VIM-2_ gene cassette of *P. stutzeri* 40D2 was confirmed within class 1 integron. The cassettes include complete CDS of 1014 bp *intI1* gene, 459 bp *aacA7* gene, 801 bp *bla*_VIM-2_ gene, 237 bp *dfrB5* gene, 477 bp *aacC-A5* gene and 575 bp *tniR* (partial). The genome mapping (4409 bp) confirmed the presence and organization of MBL specific *bla*_VIM-2_ gene between *aacA7* and *dfrB5* gene (Figure 1) comparable to the VIM-2 cassette of *P. aeruginosa* 7052; however, distinct from that of other *Pseudomonas* spp. (Figure 1). The studied class 1 integron structure of 4409 bp, is named as In559 by INTEGRALL (http://integrall.bio.ua.pt/?acc=KT716347). The location of this CDS in chromosomal DNA has been identified through whole genome sequencing of 40D2 (accession number: MWUI00000000; contig 32 and 88). The *aacA7* gene encodes aminoglycoside acetyltransferase, which confers resistance to aminoglycosides, but in our study *P. stutzeri* 40D2 was susceptible towards amikacin, an aminoglycoside. *aacA7* gene encoded enzyme belongs to AAC (6’)-I subfamily ^14^ which is significant for amikacin resistance in *P. aeruginosa*; however, variants of this enzyme fail to provide amikacin resistance in clinical isolates.^15, 16^ The unusual amikacin sensitivity in *P. stutzeri* 40D2 might be due to the presence of enzyme variants or accumulation of amikacin in bacterial cell due to alteration of OprD or efflux pump or other unknown mechanism that needs to be assessed further. Gentamicin resistance marker *aacC-A5* encoding gentamicin 3'-acetyltransferase was found immediately downstream of *dfrB5*-encoding dihydrofolate reductase, which confers resistance to trimethoprim.

In summary, we present the first known worldwide report of the chromosomal located class 1 integron containing *bla*_VIM-2_ carbapenemase gene cassette embedded in *tniR* in *P. stutzeri*. Moreover, this is also the first known report of a VIM-2 producing *P. stutzeri* in Bangladesh. We further commence to perform complete genome sequencing of the retrieved isolate to reveal the inheritance of other mobile genetic elements and resistant gene markers. This investigation concludes that emergence of *intI1* associated *bla*_VIM-2_ gene cassette-mediated carbapenem resistance in opportunistic pathogen *P. stutzeri* demands a serious public health concern because this gene cassette is considered as a xenogenetic pollutant causing acquisition of foreign resistant gene mostly in Gram-negative bacteria.

## Ethical approval

The patient was aware of his involvement in this study and gave his consent to participate in this research work. This study was reviewed by ethical committee of faculty of Biological Science, University of Dhaka, Bangladesh.

## Figures and Tables

**Figure 1:**
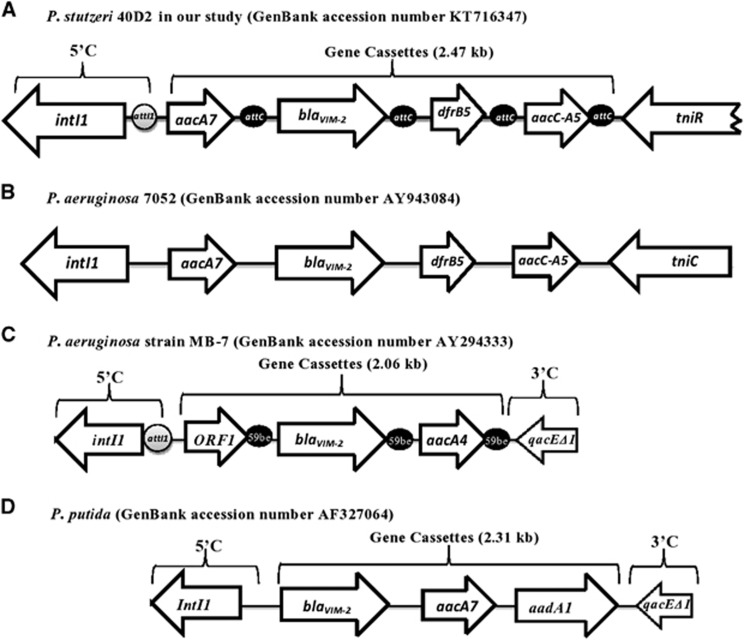
Diversity of class 1 integron that contains the *bla*_VIM-2_ gene cassettes from *Pseudomonas* spp. (A–D) *Pseudomonas stutzeri* 40D2, has been retrieved in our study. The 5′CS contains the *intI1* integrase gene. Inserted gene cassettes and their transcriptional orientations are indicated by arrows. The *attI1*and *attC* recombination sites are represented by a gray circle and black circles, respectively.
