# Supplementary material for: Emergence of IntI1 associated blaVIM-2 gene cassette-mediated carbapenem resistance in opportunistic pathogen Pseudomonas stutzeri
Source: Emerg Microbes Infect. 2017 May 10;6(5):e29–. doi: 10.1038/emi.2017.12 (PMC5520477; doi:10.1038/emi.2017.12)
Supplement: Supplementary Table S1 [file emi201712x1.pdf]

Supplementary Table, S1: Primers used to retrieve class 1 integron encoded MBL gene cassette.

Primers	Sequence (5′→ 3′)	References
VIM2F	5'-ATTGGTCTATTTGACCGCGTC-3'	[1]
VIM2R	5'-TGCTACTCAACGACTGAGCG-3'	
NDM1F	5'-GGTTTGGCGATCTGGTTTTC-3'	[2]
NDM1R	5'-CGGAATGGCTCATCACGATC-3'	
IMP3F	5'-CGGATGAAGGCACGAAC-3'	[3]
IMP3R	5'-AAGCAGACTTGACCTGA-3'	
IMP4F	5'-ATGAGCAAGTTATCTGTATTCT-3'	[4]
IMP4R	5'-AGTGTGTCCCGGGCCACC-3'	
CS 5	5'-GGCATCCAAGCAGCAAG-3'	[5]
Inti1_F	5' -GGCCATCAGCGGACGCAGGG--3'	This study
Inti244_R	5' GACATTCGAACCGTGCAG-3'	
Intii11_F	5' GGACGCAGGGAGGACTTT-3'	This study
Intii329_R	5'-CGTAGAACAAGCAGGCATCA-3'	
INTF1	5'-CGCTGCCCTACC TCTCACTA-3'	This study
INTR1	5'-TCTGCGATCTCTGACTGGTG-3'	
INT 1492 R1	5'- GCAAACCCTAGTGCTTCTCC -3'	This study
574-573F1	5'-AGATTGTCGGGTGGTACTGC-3'	This study
574-1147R1	5'-TGCGCCTATAGCCTTGAGTT-3'	
523- 70F1	5'-TCTGCGAGTGTGCTCTATGG-3'	This study
523- 592R1	5'-GCAGTACCACCCGACAATCT-3'	
643-183F1	5'-ACAACACTACCCGGAAGCAC-3'	This study
643-825R1	5'-TTCGACTGACACTCGTCTGC-3'	
533E-R1	5'-TGCGAAGAACTCGGGATTAC-3'	This study
533-F1	5'-AATTCGAGCAGCAAAGAAGC-3'	

1. Lee K, Lim JB, Yum JH et al. *bla*$_{VIM-2}$ cassette-containing novel integrons in metallo-beta-lactamase-producing *Pseudomonas aeruginosa* and *Pseudomonas putida* isolates disseminated in a Korean hospital. *Antimicrob Agents Chemother* 2002; **46**: 1053-8.

2. Nordmann P, Poirel L, Carre¨r A et al. How To Detect NDM-1 Producers. *J Clin Microbiol* 2011; **49**: 718-21.

3. Iyobe S, Kusadokoro H, Ozaki J et al. Amino acid substitutions in a variant of IMP-1 metallo-beta-lactamase. *Antimicrob Agents Chemother* 2000; **44**: 2023-7.

4. Chu YW, Afzal-Shah M, Houang ET et al. IMP-4, a novel metallo-beta-lactamase from nosocomial Acinetobacter spp. collected in Hong Kong between 1994 and 1998. *Antimicrob Agents Chemother* 2001; **45**: 710-4.

5. Levesque C, Piche L, Larose C et al. PCR mapping of integrons reveals several novel combinations of resistance genes. *Antimicrob Agents Chemother* 1995; **39**: 185-91.

.
